# Focused Ultrasound-Induced Blood–Brain Barrier Opening to Enhance Temozolomide Delivery for Glioblastoma Treatment: A Preclinical Study

**DOI:** 10.1371/journal.pone.0058995

**Published:** 2013-03-19

**Authors:** Kuo-Chen Wei, Po-Chun Chu, Hay-Yan Jack Wang, Chiung-Yin Huang, Pin-Yuan Chen, Hong-Chieh Tsai, Yu-Jen Lu, Pei-Yun Lee, I-Chou Tseng, Li-Ying Feng, Peng-Wei Hsu, Tzu-Chen Yen, Hao-Li Liu

**Affiliations:** 1 Department of Neurosurgery, Chang-Gung University and Memorial Hospital, Taoyuan, Taiwan; 2 Department of Electrical Engineering, Chang-Gung University, Taoyuan, Taiwan; 3 Department of Biological Sciences, National Sun Yat-sen University, Kaohsiung, Taiwan; 4 Department of Nuclear Medicine and Molecular Imaging Center, Chang-Gung University and Memorial Hospital, Taoyuan, Taiwan; 5 Healthy Aging Research Center, Chang-Gung University, Taoyuan, Taiwan; The Ohio State University, United States of America

## Abstract

The purpose of this study is to assess the preclinical therapeutic efficacy of magnetic resonance imaging (MRI)-monitored focused ultrasound (FUS)-induced blood-brain barrier (BBB) disruption to enhance Temozolomide (TMZ) delivery for improving Glioblastoma Multiforme (GBM) treatment. MRI-monitored FUS with microbubbles was used to transcranially disrupt the BBB in brains of Fisher rats implanted with 9L glioma cells. FUS-BBB opening was spectrophotometrically determined by leakage of dyes into the brain, and TMZ was quantitated in cerebrospinal fluid (CSF) and plasma by LC-MS\MS. The effects of treatment on tumor progression (by MRI), animal survival and brain tissue histology were investigated. Results demonstrated that FUS-BBB opening increased the local accumulation of dyes in brain parenchyma by 3.8-/2.1-fold in normal/tumor tissues. Compared to TMZ alone, combined FUS treatment increased the TMZ CSF/plasma ratio from 22.7% to 38.6%, reduced the 7-day tumor progression ratio from 24.03 to 5.06, and extended the median survival from 20 to 23 days. In conclusion, this study provided preclinical evidence that FUS BBB-opening increased the local concentration of TMZ to improve the control of tumor progression and animal survival, suggesting its clinical potential for improving current brain tumor treatment.

## Introduction

At least 18,000 patients are diagnosed with malignant primary brain tumors in the United States each year and more than half of them have glioblastoma multiform (GBM), making it the most common malignant brain tumor in adults [1]. Currently, the most common approach to identify GBM in-vivo is based on detecting the leakage of dye into regions where the tumor has caused breakdown of the blood-brain barrier (BBB) using contrast enhanced magnetic resonance imaging (MRI), computed tomography (CT) or nuclear imaging. However, such contrast-enhanced areas only partially represent the tumor-cell distribution and autopsy studies have demonstrated glioblastoma cells at great distances from the enhancing regions of tumors [2], [3]. Despite the generally leaky nature of the vasculature of gliomas, new vessels maintain some BBB properties that contribute to inefficient delivery of dye and drugs. Moreover, tumor-associated BBB disruption is highly heterogeneous, with the tumor core often being the most permeable compared to the impermeable proliferating tumor periphery [4]–[9]. Permeability does not necessarily correlate with tumor histology, size, or anatomical location.

Chemotherapy is an import treatment modality for GBM. In the United States, the most common systemically–administered adjuvant chemotherapeutic drugs are carmustine (1,3-bis(2-chloroethyl)-1-nitrosourea, or BCNU; molecular weight = 214 Da), procarbazine (PVC) and Temozolomide (8-carbamoyl-3-methyl-imidazo-(5,1-d)-1,2,3,5-tetrazin-4-(3H)-one; SCH 52365; TEMODA™TMZ; molecular weight = 194 Da). Carmustine is a nitrosourea drug that has been prescribed for adjuvant use, yet it has not shown significant survival benefits compared to radiotherapy alone in randomized phase III clinical trials. In contrast, TMZ is an alkylating agent of the imidazotetrazine series that possesses strong antineoplastic activity against high grade glioma such as recurrent GBM and anaplastic astrocytoma, both characterized as aggressive brain cancers [10], [11]. TMZ and a structurally related compound 5-(3,3′ -N,N′ -dimethyltriazen-1-yl)-imidazole-4-carboxamide (DTIC) exert their antitumor activity by being irreversibly converted to the linear triazine 5-(3-methyltriazen-1-yl) imidazole-4-carboxamide (MTIC). Conversion of DTIC to MTIC requires the action of cytochrome P450 in liver, whereas the conversion of TMZ to MTIC occurs by a non-enzymatic degradation process at physiological pH. MTIC is believed to be the major antitumor effector due to its potent alkylating activity [12].

Two major large scale phase III clinical trials have demonstrated the efficacy of TMZ as an adjuvant treatment for GBM. In a two-armed trial with 573 patients by the European Organization for Research and Treatment of Cancer (EORTC) the median survival of patients treated by radiation alone compared to radiation plus TMZ treatment was 12.1 vs 14.6 months, and two/five year survival improved from 10.9%/1.9% to 27.2%/9.8% [13], [14]. The New Approaches to Brain Tumor Therapy Consortium (NABTT) announced a similar improvement of survival by the combined use of TMZ during the GBM treatment phase [15] (median survival of 16.2 months and two year survival of 20%). This improvement in treatment efficacy has made the combined use of TMZ a standard treatment for patients newly diagnosed with GBM. However, the improvement and overall success remains limited and far from satisfactory in comparison to the treatment and management of other tumors. One potential strategy to improve treatment efficacy is the development of local or targeted drug delivery techniques to maximize the local drug concentration with a systemic dose of chemotherapeutic agent within the limits tolerated by the body.

Focused ultrasound (FUS) stimulation in combination with IV-injected microbubbles has recently been shown to open the blood-brain barrier (BBB) to increase plasma-to-tissue permeability, thus presenting a new opportunity for local drug delivery to brain tumors [1], [16]–[19]. Moreover, this BBB disruptive effect was found to be temporary and reversible (with the half-life of BBB permeability of 2–3 hours [20]), without damaging neural cells [16], [17]. The intravenous administration of microbubbles allows for selective disruption of the BBB by a significantly reduced exposure to ultrasonic energy and decreases the influence on the parenchyma thus minimizing cellular damage [19]. Compared to alternative approaches such as modified lipophilic chemicals or carotid infusion of hypertonic solution [8], [9], FUS thus presents a competitive and attractive alternative for local induction of BBB disruption to increase the local concentrations of chemotherapeutic agents in GBM. Enhanced carmustine delivery by FUS-BBB opening has been confirmed to increase the efficacy of GBM treatment [21]. Thus we hypothesized that local enhancement of TMZ deposition in the tumor site driven by FUS-BBB opening technology could potentially also improve treatment efficacy in a GBM animal model.

Here we investigated the therapeutic use of MRI-monitored FUS-induced BBB-disruption to enhance TMZ treatment efficacy in GBM rat models. We present evidence that MRI-monitored FUS can be beneficial for increasing the local deposition of chemotherapeutic agent, thus improving the therapeutic efficacy including tumor shrinkage and animal survival.

## Materials and Methods

### 9L Glioma animal model

9L rat glioma cells were cultured at 37°C in a humidified 5% CO_2_ atmosphere in minimum essential medium (MEM) supplemented with 10% fetal bovine serum and 1% penicillin/streptomycin (Invitrogen). Cells were harvested by trypsinization, washed once with phosphate-buffered saline (PBS), and resuspended (1×10^5^ cell/ml) in MEM for implantation into the striatum of rat brains.

Pathogen-free male Fischer 344 rats (∼180 g, 7∼8 weeks old) were purchased from the National Laboratory Animal Center (Taipei, Taiwan). To implant the 9L tumor cells, animals were anesthetized with 3% isoflurane gas and immobilized on a stereotactic frame. A sagittal incision was made through the skin overlying the calvarium, and a small dental drill was used to create a hole in the exposed cranium 0.5 mm anterior and 3 mm lateral to the bregma. Five microliters of 9L glioma cell suspension were injected at a depth of 4.5 mm from the brain surface to brain. The injection was performed over a 10-minute period, and the needle was withdrawn over another 2 minutes. The growth of tumor in the rat brain was monitored by MRI 7 days post tumor cell implantation. The overall successful rate of tumor implantation is about 86% (see Fig. S1; typical tumor progressions from histological observations see Fig. S2).

### Focused Ultrasound Treatment

**Figure 1:**
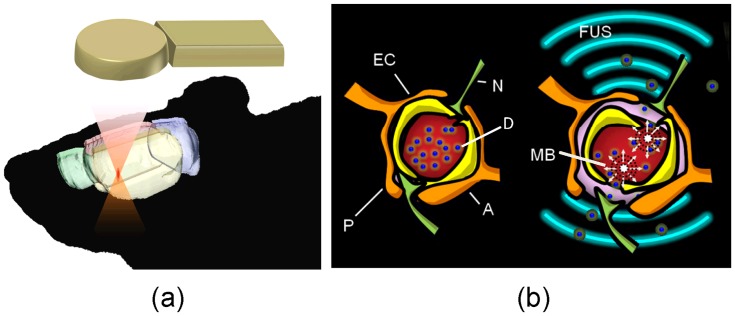
Conceptual diagrams of focused ultrasound induced blood-brain barrier opening to enhance chemotherapeutic agent delivery for brain tumor treatment. (a) Focused ultrasound is used to transcranially steer the exposure to the targeted brain tumor region; (b) focused ultrasound induces a local and reversible increase in BBB permeability of tight junctions in cerebral vessels and capillaries in the tumor core/ peripheral region. EC = endothelial cell, N = neuron, P = pericytes, A = astrocyte, D = chemotherapeutic agent, MB = microbubble.

Figure 1 illustrates the concept of FUS-induced BBB opening. A FUS transducer (Imasonics, Besancon, France; diameter = 60 mm, radius of curvature = 80 mm, frequency = 500 kHz) was used to generate concentrated ultrasound energy (Fig. 1(a)). An arbitrary-function generator (33120A, Agilent, Palo Alto, CA; and DS345, Stanford Research Systems, Sunnyvale, CA) was used to produce the driving signal, which was fed to a radio frequency power amplifier (150A100B, Amplifier Research, Souderton, PA) operating in burst mode. Animals were anesthetized by intraperitoneal injection of chlorohydrate (30 mg/kg). The top of the cranium was shaved with clippers, and a PE-50 catheter was inserted into the tail vein. The animal was placed directly under an acrylic water tank (with a window of 4×4 cm^2^ at its bottom sealed with a thin film to allow entry of the ultrasound energy) with its head attached tightly to the thin-film window. The animal head were fixed by using a self-designed stereotactic frame, and the focal beam was aligned manually by the operator [21] (geometrical relationship see Fig. S3). SonoVue® SF6-coated ultrasound microbubbles (Bracco Diagnostics Inc., Milan, Italy) were administered intravenously before treatment (0.1 mL/kg bolus of microbubbles mixed with 0.2 mL of saline). The tumor-implant hemisphere brain site was then exposed to burst-tone mode ultrasound to locally open the BBB (acoustic power = 3W; peak negative pressure = 0.6 MPa after taking into account the rat-skull inserted pressure loss of 25%; burst length = 10 ms; pulse repetition frequency = 1 Hz; exposure time = 60 s). We hypothesized that the temporary disruption of tight junctions in brain capillaries enhanced by FUS would promote a gain in TMZ permeability locally in the brain parenchyma (Fig. 1(b)).

### Animal Experiment Design

All animal experiments were approved by the Animal Committee of Chang Gung University and adhered to the experimental animal care guideline. A total of 85 animals were used, including normal (n = 30) and tumor animals (n = 55). Experiments were divided into three groups. In group 1, the aim was to confirm the FUS-BBB opening effect from leakage of small-molecule dye observed by MRI. Four subgroups included: (1) normal (n = 10); (2) normal rats with FUS-BBB opening (n = 10); (3) tumor (n = 7); and (4) tumor with FUS-BBB opening (n = 4). BBB-opening after FUS was confirmed by contrast-enhanced T1 MRI. In addition, Evans blue (EB) dye (molecular weight = 960 Da) was intravenously injected into the animals, and the amount of EB deposited in the brain was quantified spectrophotometrically (procedure described below).

In experimental group 2, the aim was to quantify the concentration of TMZ (Active Pharmaceutical Ingredients of TAMOS obtained from Lotus Pharmaceutical Co. Ltd, TAIWAN, a generic drug of Temozolomide, Schering-Plough, NJ, USA; 10% Dimethyl sulfoxide (DMSO) were used as a solvent of the TMZ powder and orally administered by gavage) by LC-MS/MS analysis in the tumor-bearing animals. Previous preclinical study reported that TMZ eliminates rapidly with a short half life of 1.2 hours in rats, gender independent, and the absolute oral availability can reach 96% [22]. Normal animals were divided into two groups: (1) TMZ oral delivery (100 mg/kg; n = 4); and (2) TMZ oral delivery following FUS-BBB opening (100 mg/kg; n  = 6). Cerebrospinal fluid (CSF; total of 60 µL and divided into 3 samples per each animal) and plasma (total of 450 µL and divided to 3 samples per each animal) were analyzed. These doses were selected based on high correlation with the human dosing regimen and has been typically applied in rodent glioma model [23], [24].

**Figure 2:**
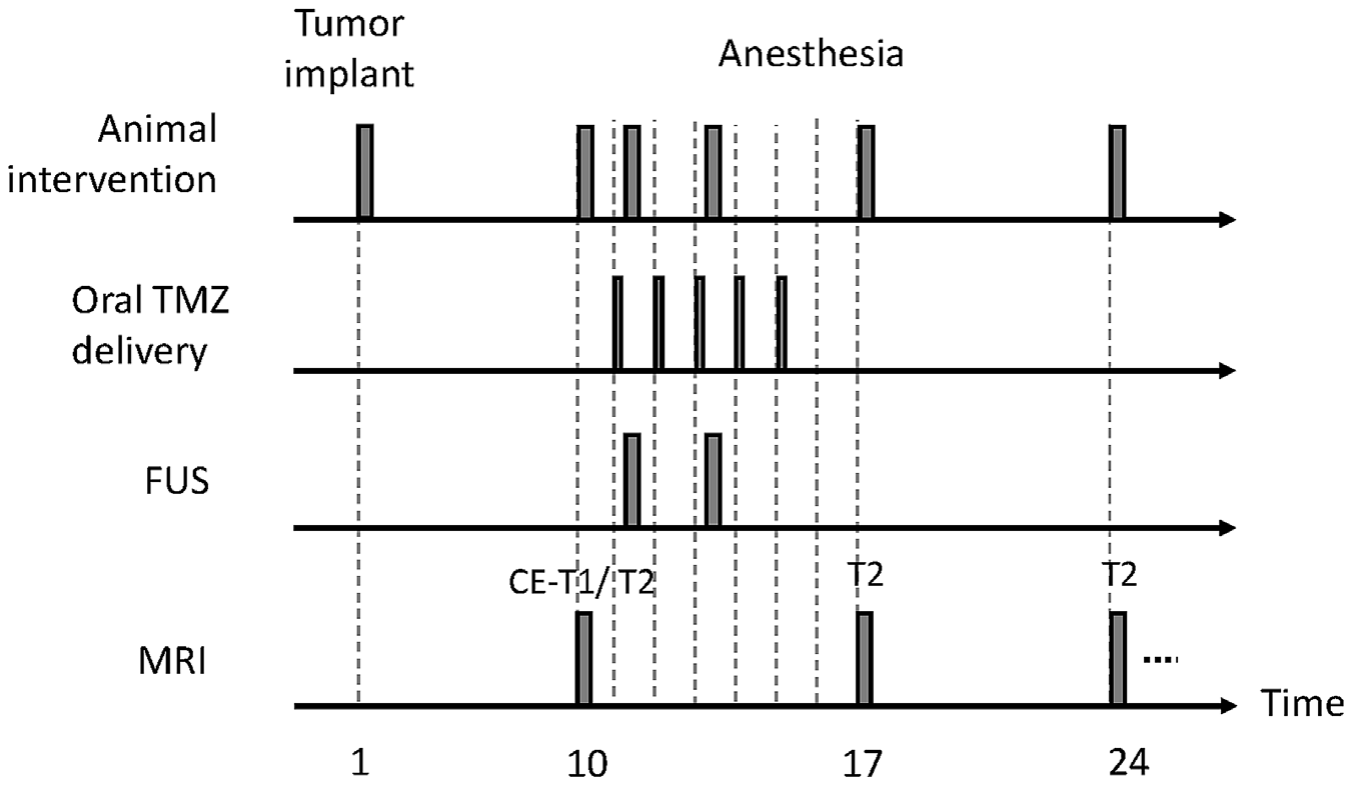
Time course for animal experiments and longitudinal MRI follow-up in experimental group 3.

In experimental group 3, the aim was to confirm the treatment efficacy of combined FUS-BBB opening and TMZ delivery. Animals were divided into 5 subgroups: (1) sham control (n  = 7); (2) low TMZ dose oral delivery (50 mg/kg per day, 5 days total; n  = 8); (3) median TMZ dose oral delivery (75 mg/kg per day, 5 days total; n  = 10); (4) high median TMZ dose oral delivery (100 mg/kg per day, 5 days total; n  = 10). In subgroup (5), animals not only received a median dose of TMZ (75 mg/kg per day, 5 days total), but also underwent two FUS-BBB opening procedures (day 1 and day 3 immediately after TMZ administration; n  = 9;). CE-T1 weighted MR images were acquired to confirmed the BBB-opening, and tumor volume was followed by T2-weighted MRI with a 7-day period, with the tumor volume as well as animal survival analyzed. The detailed experimental timeline is demonstrated in Figure 2.

### Spectrophotometric quantitation of Evans Blue dye

EB (2% in saline) was injected intravenously (2 mg/kg) and the animals were sacrificed two hours later. All animals were first deeply anesthetized with 10% chloral hydrate and infused with heparinized saline through the cardiac ventricle until colorless infusion fluid was obtained from the atrium. After the rats had been sacrificed by decapitation, the hemispheres of the brain were separated along the transverse suture. Then both hemispheres were weighed and placed in formamide (1 ml/100 mg) at 60°C for 24 h. The sample was centrifuged for 20 min at 14,000 rpm. The concentration of dye extracted from each brain was determined spectrophotometrically at 620 nm and was compared with a standard graph created by recording optical densities from serial dilutions of EB in 0.9% sodium chloride solution. The EB tissue content was quantified via a linear regression standard curve derived from seven concentrations of the dye and is shown as the amount of dye per gram of tissue.

### Magnetic Resonance Imaging and Analysis

All MRI images were acquired on a 7T scanner (Trio with Tim, Siemens, Erlangen, Germany) using the standard wrist coil with an inner diameter of 13 cm. In the normal animal experimental group, FUS-induced BBB opening was monitored by MRI with a 7-Tesla magnetic resonance scanner (Bruker ClinScan, Germany) and a 4-channel surface coil. The animals were anesthetized through inhalation of 2% isoflorane throughout the MRI process, placed in an acrylic holder and positioned in the center of the magnet. An intravenous bolus (0.1 mmol/kg) of gadopentetate dimeglumine MRI contrast agent (Magnevist, Berlex Laboratories, Wayne, NJ) was administered before scanning. The tumor location and region of FUS-induced blood–brain barrier disruption were determined by performing a gradient echo FLASH sequence to acquire T1W images with the following imaging parameters: pulse repetition time (TR)/echo time (TE) = 300/3.81 ms; FOV = 21×25 mm^2^; in-plane resolution = 0.25 ×0.2 mm^2^; slice thickness = 0.5 mm; flip angle  = 70°. In the tumor animal experiment group, tumor size was quantified using turbo-spin-echo based T2-weighted images with the following parameters: pulse repetition time (TR)/echo time (TE) = 2440/37 ms; FOV = 34×40 mm^2^; in-plane resolution = 0.4×0.3 mm^2^. Animals in experimental group 3 were followed up to monitor the evolution of brain tumors. The relative tumor size was estimated by summing all voxels (i.e., 0.25×0.2×0.5mm^3^/voxel) that represents tumor regions from every T2-image slide, and animals were longitudinally imaged every 7 days up to 40 days.

### Quantization of TMZ

The blood level of TMZ in rats was monitored according to the methods for analysis of plasma TMZ reported by Baker et al. [12] and Portnow et al. [25]. Rats were fed with TMZ (75 mg/kg). Within 15 minutes after FUS-BBB opening procedure,, animals were euthanized with injection of an overdose of equithesin. Once the rat lost its tail pinch reflex, blood was collected by transcardiac puncture into a prechilled, heparinized syringe, immediately placed in prechilled, heparinized tubes and rapidly centrifuged at 4°C. A 50 µL of 2.5 M HCl was added to each milliliter of plasma. Standard solutions of 0.1–10 µg/mL were prepared in blank rat plasma with HCl. A 20 µL aliquot of plasma sample, standard, or control (i.e., plasma containing no TMZ) was added to 80 µL of methanol (MeOH) with internal standard (IS; 2 µg/mL of trimethyl ^13^C_3_-labelled caffeine in MeOH) and vigorously vortexed for 10 seconds, then centrifuged at 14,000 rpm for 5 minutes at 4°C. A 20 µL aliquot of the supernatant was then mixed with 300 µL of 0.5% acetic acid (HOAc) in an HPLC sample vial for subsequent LC-MS/MS analysis.

TMZ in rat CSF was collected and analyzed after euthanizing rats with injection of an overdose of equithesin. Within 30 minutes after FUS-BBB opening procedure, CSF was aspirated through the Foramen Magnum into a syringe containing 6% HOAc and transferred to a vial prechilled on ice and containing additional HOAc such that the final volume ratio of 6% HOAc:CSF was 1∶5. The mixture was briefly vortexed and centrifuged at 14,000 rpm for 5 minutes at 4°C. Twelve microliters of supernatant was mixed with 10 µL IS (500 ng/mL in MeOH/0.5% HOAc (50∶50)) and 100 µL of 0.5% HOAc in the HPLC sample vial for subsequent LC-MS/MS analysis. Standard concentrations for the analysis of TMZ in CSF were prepared over the range of 0.5 –150 ng/ml in 0.5% HOAc.

The LC-MS/MS system consisted of a Waters 2695 separation module for HPLC with an outflow that was coupled to the electrospray ionization source of an amaZon X ion trap mass spectrometer (Bruker Daltonics). TMZ and IS were eluted from an Ascentis Express C18 column (2.1×50 mm; particle size 2.7 µm) with an isocratic mobile phase (14% acetonitrile and 0.1% formic acid) at a flow rate of 0.2 mL/min. The temperature of the column was maintained at 30°C, whereas the temperature of the autosampler was kept at 5°C. The mass spectrometer was operated in positive ion mode. TMZ and IS were detected by multiple-reaction-monitoring (MRM). The transition from precursor to product ion for TMZ occurs from m/z 194.9 to m/z 137.8, and from m/z 197.9 to m/z 139.9 for IS. Each chromatography run took approximately 10 minutes. A 20 µL aliquot was injected into the column for analysis of TMZ in both plasma and CSF. Quantitative analysis of TMZ was carried out with QuantAnalysis (Bruker Daltonics).

### Histological examination

Tissues were prepared for histology after in vivo MRI analysis. Histopathology was performed on 10- µm sections from paraformaldehyde-fixed, paraffin-embedded brains. In the parametric testing group of normal animal experiments, EB dye was administered after MRI and before animal sacrifice for gross observation of the BBB disrupted region. Animals were sacrificed four hours after dye injection. Slides were placed in a staining jar containing a hydrochloric acid-potassium ferrocyanide solution for 30 minutes at room temperature. The slides were rinsed in distilled water and were counterstained by nuclear fast red for 5 min. Microscopy was performed using a Zeiss Axioplan 2 imaging microscope with AxioVision 4.1 imaging software, AxioCam HRc camera, and Zeiss objectives Fluar 10×/0.50, Plan-Apochrome 20×/0.75, and Plan-Neofluar 100×/1.30 oil (Carl Zeiss Vision, Oberkochen, Germany). Hematoxylin and eosin (H&E) staining was conducted to evaluate the ultrasound-induced brain tissue damage. For brain-tumor implant animals, H&E staining was also carried out to histologically confirm the tumor progression.

### Statistical analysis

The statistical significance of increased signal intensity was determined using a two-tailed unpaired *t* test, with *p*<0.05 considered to be significant. In experimental group 3, the Kaplan-Meier method was used to perform animal survival analysis. Statistical significance was calculated using the Mantel-Cox test, with statistical significance assumed at p<0.05. The different treatment groups were compared in terms of survival time, increase in median survival time (IST_median_) and maximal survival time.

## Results

**Figure 3:**
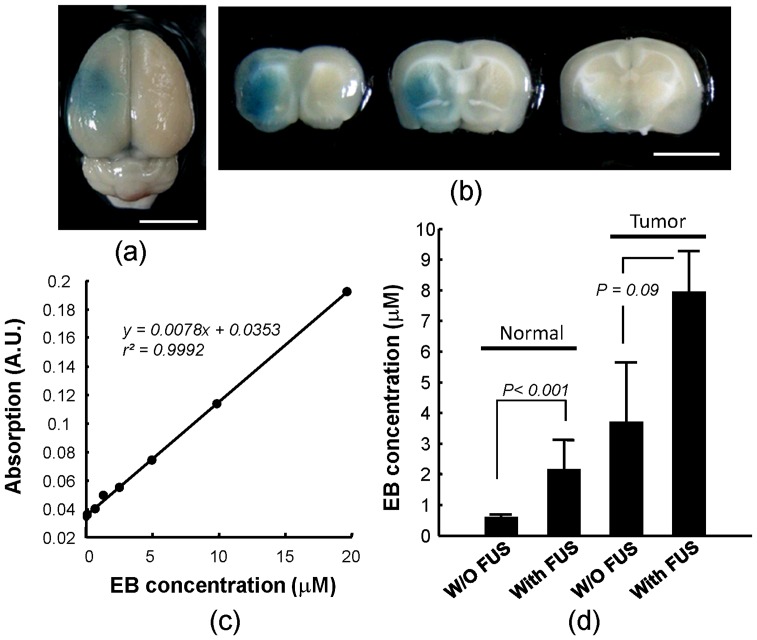
Representative Evans Blue dye stained Brain sections and calibrations after inducing FUS-BBB opening. (a, b) brain sections viewed from the top and in corresponding brain sections. Bar = 5 mm. (c) Calibration of Evans Blue dye concentration using its correlation with ELISA light absorption (r^2^ = 0.9992). (d) Evans Blue quantification of experimental group 1 animals. FUS-BBB opening reached a 3.8-fold increase in EB concentration in normal rats (p<0.001) and a 2.1-fold increase in tumor rats (p = 0.09).

The capability of FUS to disrupt the BBB in rat brain was analyzed and evaluated in experiment group 1. EB dye leakage was used to illustrate that FUS was effective at local disruption of the intact BBB in normal rats as viewed from the top of the brain (Fig. 3(a)) and in dissected brain sections (Fig. 3(b)). BBB-opening was clearly identified as EB-stained blue regions in brain parenchyma. The amount of EB increased in a highly linear manner with the detected ELISA signal as shown by the calibration curve in Figure 3(c) (r^2^ = 0.9992), thus allowing precise spectrophotometric quantitation of EB deposition in the brain.

The amount of EB deposited in the brain was compared among four experimental animal subgroups (Fig. 3(d)). In general, the FUS-treated side of the brain showed significantly higher extravasation of EB dye compared to the contralateral brain without FUS treatment, in either normal or tumor tissue. The lowest EB amount (0.56±0.09 µM) was detected in normal control animals. FUS-induced BBB-opening significantly increased the EB amount by 3.8 fold (2.13±0.99 µM; p<0.001). In tumor-bearing animals, tumor regions originally had BBB deficits so that EB dye could penetrate into the tumor region (3.67±1.97 µM). However, when enhanced by FUS, EB deposition in the brain tumor region further increased by 2.1 fold (7.92±1.36 µM), but did not reach statistical significance (p = 0.09).

**Figure 4:**
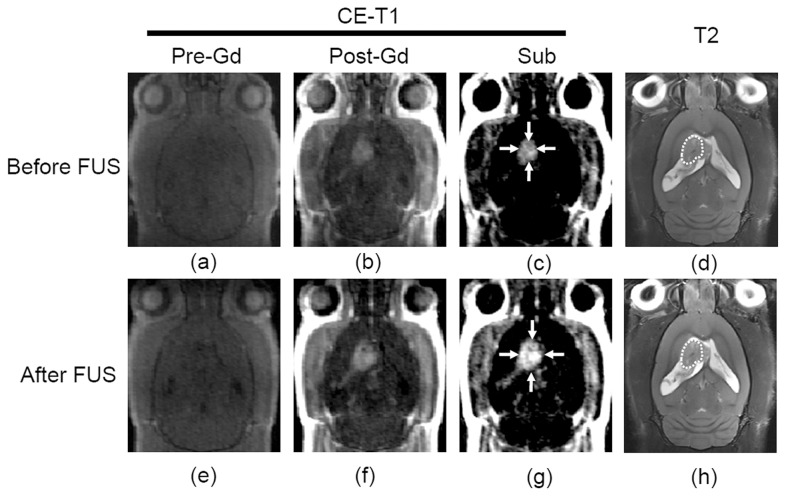
Representative MR images before (upper panel) and after (lower panel) conducting FUS BBB-opening in rat brain tumors. (a, e) T1-weighted images; (b, f) Gd-DTPA contrast-enhanced T1-weighted images; (c, g) subtracted after and before Gd-DTPA injection T1 images; (d, h) T2-weighted images.

Figure 4 demonstrates a typical example of the effect of FUS BBB-opening in the tumor region. In the absence of FUS, Gd-DTPA was capable of leaking into the tumor due to characteristic tumor-induced compromise of the BBB (Fig 4(a)-(c)). However, after being exposed to FUS, the MR signal intensity was increased and the enhanced regions expanded, indicating that FUS-BBB opening could effectively enhance Gd-DTPA permeability in the tumor (Fig. 4(e)-(g)). There was no significant local image intensity change in T2-weighted images after FUS exposure (Fig. 4(d) versus (h)), indicating that no additional tissue damage or edema had been induced at this FUS exposure, and confirming the safety of the selected pressure not only in normal brains, but also in tumor-implanted ones.

**Figure 5:**
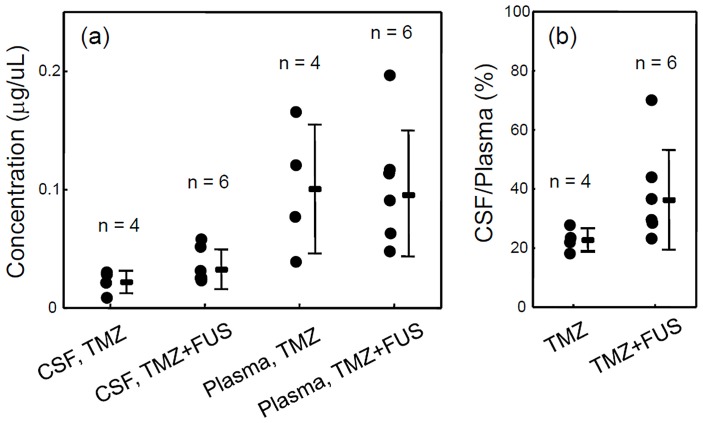
In-vivo TMZ concentration measurement. (a) Measured TMZ concentrations (in µg/ µL) in cerebrospinal fluid (CSF) and blood plasma in animals treated with TMZ only (TMZ; n = 4) or combined TMZ with FUS-BBB opening (TMZ+FUS; n = 6). (b) Corresponding CSF/Plasma ratios (in %) determined from (a) (p = 0.06).

We measured the TMZ concentration in CSF and plasma for TMZ administration only animals (n  = 4) compared to combined TMZ and FUS administration (n = 6) (experimental group 2). The TMZ concentration was similar in the plasma (Fig. 5(a); 0.1±0.055 µg/ µL and 0.096±0.053 µg/ µL respectively; no significant difference (p = 0.909)), yet in the CSF the TMZ/FUS groups had a higher average concentration (Fig. 5(a); 0.032±0.017 µg/ µL versus 0.022±0.01 µg/ µL in the TMZ group; no significant difference (p = 0.225)). When the individual TMZ levels in the CSF and plasma were expressed as ratios (Fig. 5(b)), the CSF/Plasma ratio was 22.7±3.9% in the TMZ alone group, which was similar to a previous report (23). The TMZ/FUS group, however, was found to have an elevated average level of 38.6±16.8% (p = 0.06), indicating that FUS-induced BBB-opening could indeed enhance the TMZ concentration in brain parenchyma.

**Figure 6:**
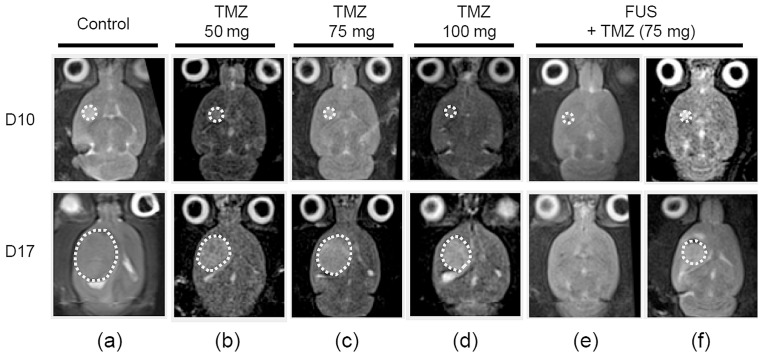
Representative T2-weighted MR images to monitor brain tumor progression at day 10 and 17 in experimental group 3. (a) sham control; (b) low dose TMZ oral delivery; (c) median dose TMZ oral delivery; (d) high median dose TMZ oral delivery; (e, f) median dose TMZ integrated with FUS-BBB opening procedures.

**Figure 7:**
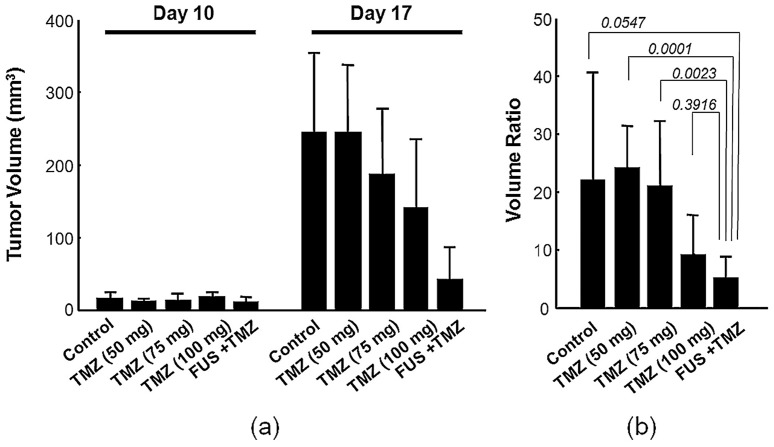
Tumor progression observation among groups. (a) Tumor volume (in mm^3^) between day 10 and 17 in experimental group 3; (b) Ratio of the tumor volume between day 17 and day 10 determined from (a).

In the third animal experiment group, we aimed to evaluate whether the gain in TMZ deposition due to FUS-induced BBB opening could improve glioma treatment outcome. Control of tumor progression by different treatment protocols was assessed over time by T2-MRI. Typical tumor follow-up images are shown in Figure 6 (days 10 and 17), and were quantified in Figure 7. First, the null hypothesis check among these experimental groups to confirm the rejection of the null hypothesis (p<0.05 in both ANOVA test and Wiscoxon rank sum test; see Table S1). Overall, pair-wise comparison among the experimental groups found TMZ/FUS group had significant difference with others except the high-dose TMZ group; see Table S2). Control tumor animals and low/medium dose TMZ oral delivery showed the poorest outcome for controlling tumor progression among the subgroups (Fig. 6(a)- 6(c)). Tumor progression ratios (defined as the ratio between the tumor volume measured in day 17 and day 10) in these three groups were 24.03±7.35 (low dose), and 20.96±11.2 (medium dose) compared to 22.03±18.6 (control), whereas high dose TMZ oral delivery appeared to have an average but not significant tumor suppression effect (Fig. 6(d); 9.16±6.79). The animals that received a medium TMZ dose together with FUS exposure showed a more definitive tumor progression control effect; either overall tumor shrinkage (Fig. 6(e)), or relatively better tumor-progression control (Fig. 6(f)) compared to the high dose TMZ group (5.06±3.78; p = 0.0547, 0.001 and 0.023 respectively when compared with control, low-TMZ, and medium-TMZ group).

**Figure 8:**
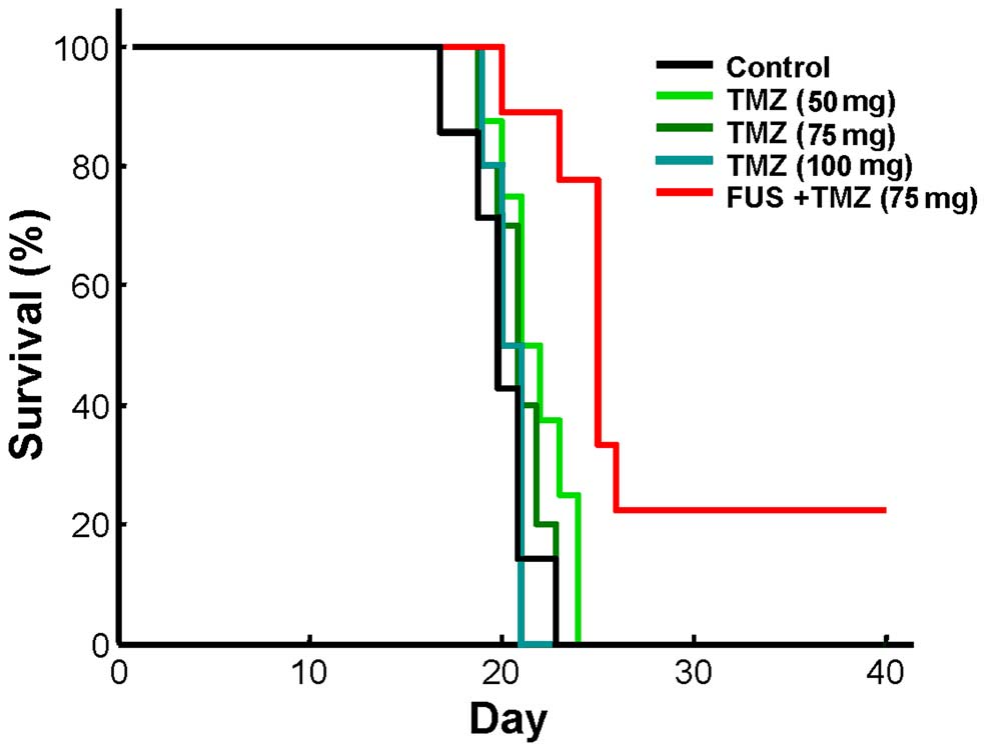
Kaplan–Meier plot demonstrating animal survival in experimental group 3.

Next we compared animal survival among the groups (Fig. 8; Table 1). Although high dose TMZ delivery seemed to control tumor progression, oral delivery of TMZ did not effectively extend animal survival time. Strikingly, only integrated TMZ delivery and FUS-BBB opening significantly prolonged the median survival when compared to control, with the IST_median_ showing a 15% increase (Table 1). Of note, two of the TMZ+FUS treated animals remained alive after 40 days, and the mean survival time increased to 26.3±8.0 days, showing a 37.7% increase compared to the mean survival time in the control group.

**Table 1:** Efficacy of various treatment protocols of induced brain tumors in rats.

Group (n*)	Median survival(days)	IST_median_ (%)	Mean survival (days)	Maximal survival (days)	*p value*	Hazard ratio (95% CI)
Control (7)	20	—	19.1±1.9	22	—	1.0
TMZ, 5 mg (8)	20.5	2.5	20.7±1.8	23	0.0891	0.390 (0.131–1.155)
TMZ, 7.5 mg (10)	20	0	20.1±1.5	22	0.4305	0.677 (0.256–1.787)
TMZ, 10 mg (10)	19.5	−2.5	19.3±0.8	20	0.8123	1.127 (0.421–3.019)
TMZ+FUS (9)	23	15	26.3±8.0	>40‡	0.0009	0.111 (0.030–0.409)

Increase in median survival time (IST_median_), *p* value, hazard ratio, and 95% confidence interval are relative to the control group (Analysis with using TMZ+FUS group as reference is shown in the Table S3).

* n = Number of animals per group.

† Represented in mean±S.D.

‡ Two animals were still alive after day 40.

**Figure 9:**
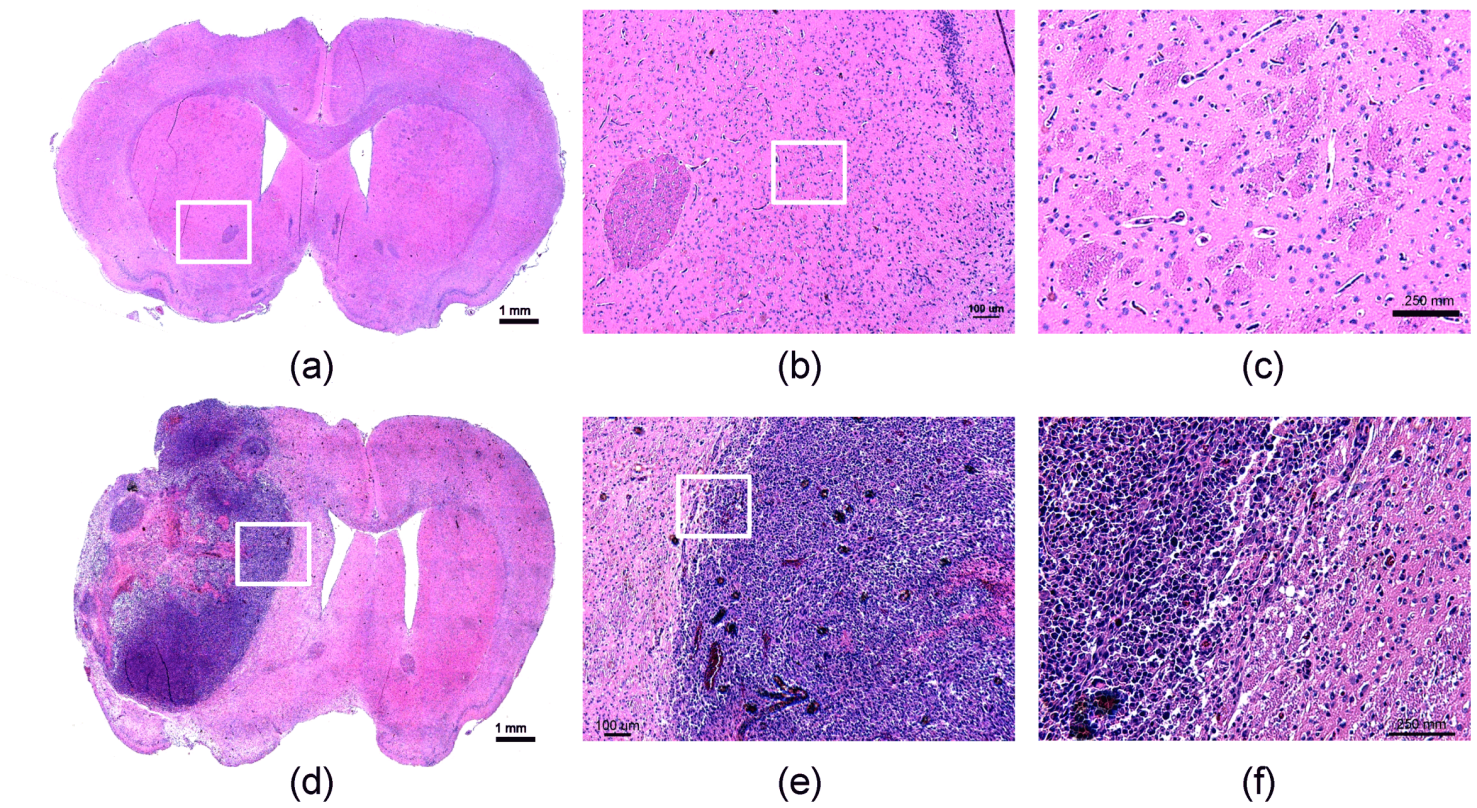
Hematoxylin and eosin (H&E) stained sections of rat brains. Tissues were collected for analysis from the TMZ-delivery alone ((a) – (c); brain samples obtained immediately after animal died) or combined TMZ/FUS exposure animals ((d) – (f): brain samples obtained after day 90). (a) and (d): whole brain section; (b) and (e): 4 ×;(c) and (f): 10 ×.

Typical H&E stains of brains obtained from animals treated by either TMZ-alone or enhanced by FUS are shown in Figure 9. In TMZ-alone animals, H&E stains showed large tumor cell populations characterized by dense nuclear distribution and mixed with necrosis within tumor regions (Fig. 9(a) – (c)). In contrast, in the successful treatment case obtained from FUS-enhanced TMZ-delivery in which tumor shrinkage was observed by MRI, we noted only tiny areas of gliosis infiltrated with chronic inflammatory cells (Fig. 9(d) – (f)).

## Discussion

Since the mechanism of FUS-induced BBB opening is to reduce the stringent selection of molecules that penetrate through the temporarily disrupted tight-junctions, any therapeutic agent could potentially be delivered. For example, in a recent NABTT phase III trial, Grossman et al. demonstrated that the combined delivery of a new chemotherapeutic agent, Poly-ICLC (Oncovir), together with standard radiation/TMZ treatment can prolong median survival from 16.2 to19.6 months, and the two-year survival rate can increase from 27% to 37% [15].

Large molecules could also be delivered via FUS-BBB opening. The first promising outcome in FUS-enhanced brain drug delivery was the successful enhanced delivery of liposomal-doxorubicin for preclinical brain-tumor treatment [26], [27]. The diameters of liposome-encapsulated doxorubicin particles range from 66–75 nm and pegylated modification of the lipid layers ensures long circulation times. Treat et al. reported that FUS-BBB opening enhances delivery of liposomal-doxorubicin into brain parenchyma up to a concentration of 2,369±946 ng/g tissue, which is several-fold higher than the therapeutic dose of doxorubicin treatment for breast carcinoma. We recently demonstrated in preclinical experiments that chemotherapeutic agents conjugated to biodegradable magnetic nanoparticles that were even larger than liposomal-doxorubicin (up to 90 nm) can be delivered through the opened BBB and significantly improves the animal mean survival from 18.3 to 30 days (66% increase) when combined with magnetic targeting [28].

A variety of approaches have been proposed for overcoming the resistance of GBM tumors to treatment, including intra-arterial or interstitial injection of chemotherapeutic agents [29], implantation of biodegradable matrices containing chemotherapeutic agents into the debulked tumor cavity [30], [31], convection-enhanced delivery of molecules [32], [33], and mixing with water-miscible organic solvents such as ethanol to allow delivery of high concentrations of lipid-soluble chemotherapeutic agents to the tumor. Despite promising results presented in preclinical models, clinical trials using these approaches have been less encouraging, varying from increased normal tissue toxicity to only a modest increase in patient stabilization and survival. Osmotic agents have also been used to increase permeability of the blood–tumor barrier to therapeutic agents [6], [7], [34], [35]. Clinical trials have shown that combining chemotherapy with enhanced BBB permeability can improve treatment outcome, mainly by increasing expected survival. Like the FUS method used in our study, osmotically enhanced permeability is transient. However, administration of osmotic solutions alters the BBB systemically: Its effects cannot be targeted to a specific position. In contrast, FUS retains the ability to open the BBB temporarily, but more importantly, the region to be disrupted can be limited and/or defined to conform to the needs of an individual patient, thus has potential to avoid serious side-effects of global BBB-opening approach and resulting in increased safety.

Although TMZ has potent antineoplastic activity, its other peculiar chemical features impose a challenge for quantitation and pharmacokinetic analysis. In aqueous buffers, TMZ is stable at pH<4, but it rapidly decomposes to MTIC at pH>7. MTIC, on the other hand, is stable at alkaline pH, but rapidly breaks down to AIC at pH<7 [11], [12], [36], [37]. The in vitro half-life of TMZ in phosphate buffer at pH 7.4 is 1.9 hours at 37°C compared to only approximately 2 min for MTIC at the same temperature, and up to about one hour for MTIC when the temperature is decreased to 4°C [22]. Unlike TMZ and DTIC, MTIC is only detectable when incubated with microsomes [38] at low pH. This inherent instability of MTIC has complicated its quantitation. TMZ is most commonly quantitated by high-performance liquid chromatography (HPLC) [10], [11], [22], MS/MS analysis [39] or radiolabel/radioactivity detection (23,39).

We used LC-MS/MS where the pH could be precisely controlled immediately after sample collection and preparation to successfully quantitate TMZ in CSF and plasma. Our results confirmed that FUS-induced BBB-opening could enhance the CSF/plasma ratio of TMZ in an animal model (from 22.7% to 38.6%). However, measurement of TMZ in brain tissues and other organs is currently not feasible and we note that the measured CSF/plasma ratio likely far underestimates the actual accumulation of TMZ in brain tumor tissues where the direct FUS enhancement takes place. Despite this drawback, our current established LC-MS/MS analytical technique is crucial for understanding the pharmacokinetic/pharmacodynamic behaviors of TMZ when enhanced by FUS exposure, and this technology is currently being further developed.

Although our results are promising, the use of FUS to enhance delivery of chemotherapeutic agents for glioma treatment still has some limitations. The 500-kHz FUS transducer used here allows transcranial use, but the focal size is limited to a tadpole shape with a long axis of about 20 mm and may not satisfy the need for true point-like BBB-opening. However, considering that a typical brain tumor is several centimeters long, the use of a spherical transducer may be substituted for clinical use. In addition, a hemispherical type transducer could effectively shape the dimension to be isotropic when more precise focal point control is necessary in brain tumor treatment [40]. Our current protocol does not permit the BBB-disrupted region to conform precisely to the targeted tumor. This raises the possibility of inducing off-target tissue damage caused by the release of high levels of TMZ into the surrounding normal brain. Future improvements of precise targeting may include the use of MR-guided [16], [18] or neuronavigation-guided procedures [41] to guide BBB-opening. Third, the indices of treatment efficacy only considers tumor progression and survival and may not fully reflect treatment outcome. Additional information including physiological data, changes in animal immunity, and biodistribution of chemotherapeutic agents may be necessary to conclusively demonstrate the safety and efficacy of this approach. Also, in this study we only conducted two FUS exposures out of the 5-day TMZ administration session. This protocol may not be optimized, and adding more FUS exposure could be beneficiary for further enhancing TMZ local deposition and can be further investigated in the future.

In our previous attempt of using FUS-BBB opening for the enhanced delivery of BCNU, an improved treatment efficacy was observed, which the median survival was increased to 85.9% when compared with control group [21]. On the other hand, Treat et al. also reported treatment efficacy improvement can be observed when employing FUS-BBB opening to enhance liposomal-doxorubucin (Doxil) delivery into 9L-glioma animals [26]. The reported median survival showed slightly improvement with the aid of focused ultrasound, which yields improved median survival time of 24% than control animals (from 25 to 31 days). In this study, we observed that the median survival improvement was close to what Treat et al reported (from 20 to 23 days) but were both relatively lower than our previously reported values from C6-glioma/BCNU treatment. This implies that the degree of improved treatment efficacy strongly correlates with the selected animal models (it should be also noted that both C6 and 9L glioma model do not present strong infiltrating capability and may also contains different immunogenity levels [42]), yet, this proposed technology indeed generally enhances penetration/deposition of various types of chemotherapeutic agent and provide improvement of therapeutic efficacy.

## Conclusion

Here we showed that noninvasive FUS treatment enhanced delivery of TMZ through the BBB such that the chemotherapeutic drug dosage could be increased specifically in the tumor region. FUS-enhanced delivery of TMZ significantly suppressed tumor growth and prolonged animal survival, suggesting that this approach may improve the future therapeutic outcome of brain tumor TMZ chemotherapy. Because TMZ is the first-line chemotherapeutic drug for treatment of GBM, this procedure could be highly clinically relevant, with the potential to ultimately advance the use of chemotherapy to treat patients with central nervous system malignancies. Our findings encourage further in-depth exploration of the benefits of locally increasing the concentrations of chemotherapeutic drugs for the most effective treatment of brain tumors.

## Supporting Information

Figure S1 **Tumor progression distribution of the 9L-glioma model to demonstrate the model stability.** Totally 28 animals implanting 9L cells to serve as reference for other therapeutic intervention groups during the year 2012 in our Lab. For these totally 26 animals, the overall successful rate of tumor implantation is about 86% (except 2 out our the 24 animals died before day 10 and another 2 did not progress on day-10′s MRI screening; these 4 animals were excluded from studies). Red: In this study (n  =  7); Black: Other parallel studies.
(TIF)

Figure S2 **Pathological examinations of the tumor model.** HE stains showing tumor progressions at different time points of the model employed in this study (3, 12, 17, and 24 days after 9L-cell implantation). Upper: 4×; Lower: 20×.
(TIF)

Figure S3 **Geometrical relationship of the drill hole, focused ultrasound beam, and the implant tumor from MR images.**
(TIF)

Table S1 **Null hypothesis check among the experimental groups.** We first test the null hypothesis to check its reject (p < 0.05) validity (i.e., whether mean (control)  =  mean (TMZ, 50 mg/kg)  =  mean (TMZ, 75 mg/kg)  =  mean (TMZ, 100 mg/kg)  =  mean (FUS+TMZ, 75mg/kg)). The ANOVA test and Wiscoxon rank sum test both confirmed the rejection of the null hypothesis (p  =  0.0007 and 0.0006, respectively).
(DOCX)

Table S2 **Pair-wise comparison among the experimental group.** The comparison was to check statistical difference of the (FUS+TMZ) groups to the others (only p>0.05 when comparing with the high-dose TMZ group).
(DOCX)

Table S3 **M-Cox proportional hazard model analysis when using FUS/TMZ as a reference.**
(DOCX)
