# Supplementary figures and images for: Focused Ultrasound-Induced Blood–Brain Barrier Opening to Enhance Temozolomide Delivery for Glioblastoma Treatment: A Preclinical Study
Source: PLoS One. 2013 Mar 19;8(3):e58995. doi: 10.1371/journal.pone.0058995 (PMC3602591; doi:10.1371/journal.pone.0058995)

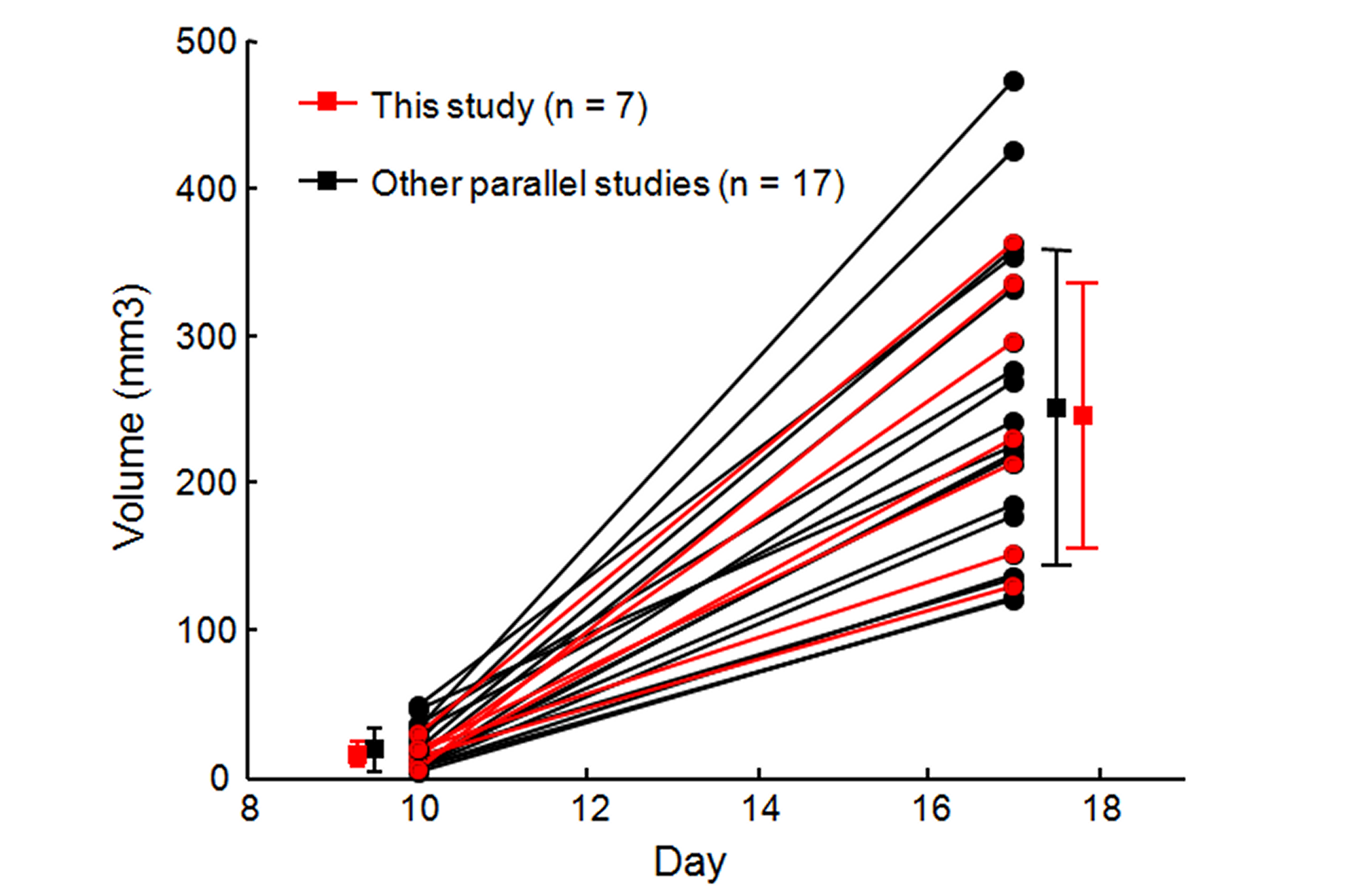

Supplement: Figure S1 — Tumor progression distribution of the 9L-glioma model to demonstrate the model stability. Totally 28 animals implanting 9L cells to serve as reference for other therapeutic intervention groups during the year 2012 in our Lab. For these totally 26 animals, the overall successful rate of tumor implantation is about 86% (except 2 out our the 24 animals died before day 10 and another 2 did not progress on day-10′s MRI screening; these 4 animals were excluded from studies). Red: In this study (n = 7); Black: Other parallel studies. (TIF) [file pone.0058995.s001.tif]

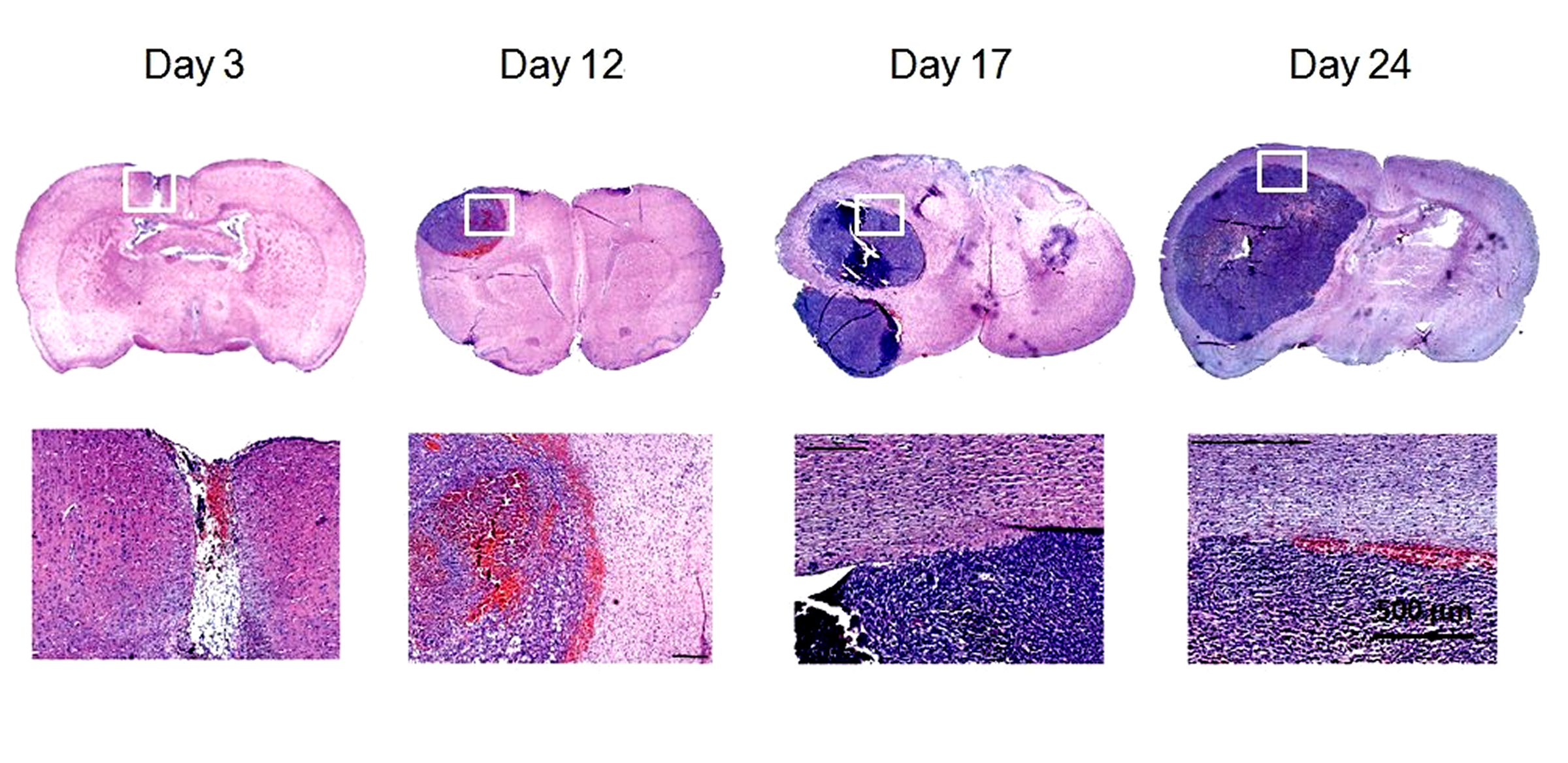

Supplement: Figure S2 — Pathological examinations of the tumor model. HE stains showing tumor progressions at different time points of the model employed in this study (3, 12, 17, and 24 days after 9L-cell implantation). Upper: 4×; Lower: 20×. (TIF) [file pone.0058995.s002.tif]

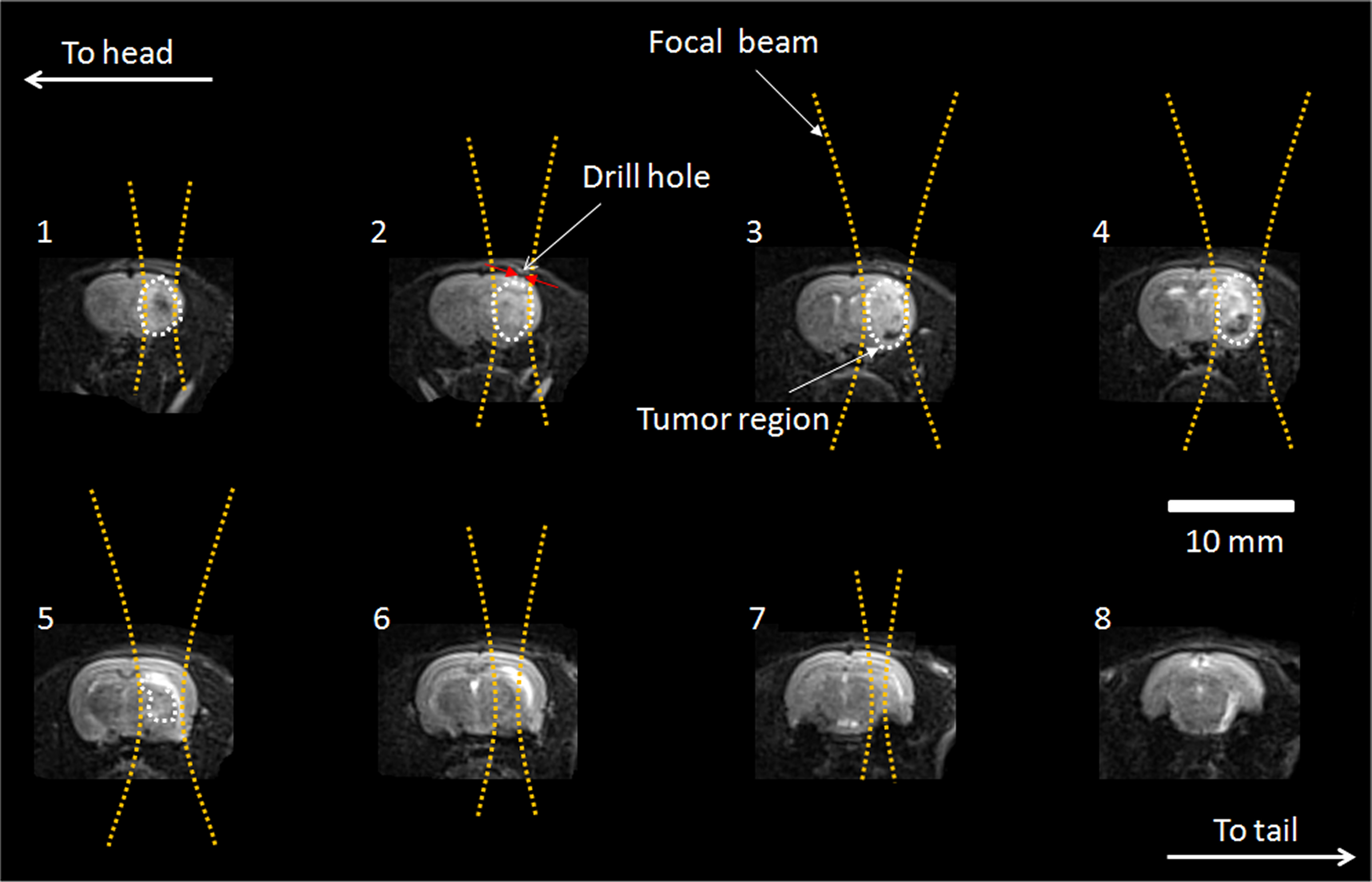

Supplement: Figure S3 — Geometrical relationship of the drill hole, focused ultrasound beam, and the implant tumor from MR images. (TIF) [file pone.0058995.s003.tif]
