# Supplementary material for: Focused Ultrasound-Induced Blood–Brain Barrier Opening to Enhance Temozolomide Delivery for Glioblastoma Treatment: A Preclinical Study
Source: PLoS One. 2013 Mar 19;8(3):e58995. doi: 10.1371/journal.pone.0058995 (PMC3602591; doi:10.1371/journal.pone.0058995)
Supplement: Table S1 — Null hypothesis check among the experimental groups. We first test the null hypothesis to check its reject (p < 0.05) validity (i.e., whether mean (control) = mean (TMZ, 50 mg/kg) = mean (TMZ, 75 mg/kg) = mean (TMZ, 100 mg/kg) = mean (FUS+TMZ, 75mg/kg)). The ANOVA test and Wiscoxon rank sum test both confirmed the rejection of the null hypothesis (p = 0.0007 and 0.0006, respectively). (DOCX) [file pone.0058995.s004.docx]

**Table S1.** **Null hypothesis check among the experimental groups.** We first test the null hypothesis to check its reject (p < 0.05) validity (i.e., whether mean (control) = mean (TMZ, 50 mg/kg) = mean (TMZ, 75 mg/kg) = mean (TMZ, 100 mg/kg) = mean (FUS+TMZ, 75mg/kg)). The ANOVA test and Wiscoxon rank sum test both confirmed the rejection of the null hypothesis (p = 0.0007 and 0.0006, respectively).

| Group | N | Ratio  (mean ± SD) | p value (ANOVA) | p value (Wilcoxon rank sum test) |
| --- | --- | --- | --- | --- |
| Control | 7 | 22.03 ± 18.60 |  |  |
| TMZ, 50 mg/kg | 8 | 24.03 ± 7.35 |  |  |
| TMZ, 75 mg/kg | 10 | 20.97 ± 11.21 | 0.0007 | 0.0006 |
| TMZ, 100 mg/kg | 10 | 9.16 ± 6.79 |  |  |
| FUS+TMZ | 9 | 5.07 ± 3.78 |  |  |
