# Supplementary material for: Focused Ultrasound-Induced Blood–Brain Barrier Opening to Enhance Temozolomide Delivery for Glioblastoma Treatment: A Preclinical Study
Source: PLoS One. 2013 Mar 19;8(3):e58995. doi: 10.1371/journal.pone.0058995 (PMC3602591; doi:10.1371/journal.pone.0058995)
Supplement: Table S2 — Pair-wise comparison among the experimental group. The comparison was to check statistical difference of the (FUS+TMZ) groups to the others (only p>0.05 when comparing with the high-dose TMZ group). (DOCX) [file pone.0058995.s005.docx]

**Table S2. Pair-wise comparison among the experimental group.** The comparison was to check statistical difference of the (FUS+TMZ) groups to the others (only p > 0.05 when comparing with the high-dose TMZ group).

| Group | p value  (Student's T test) |
| --- | --- |
| Control vs. FUS+TMZ | 0.0547 |
| TMZ 50 vs. FUS+TMZ | < 0.0001 |
| TMZ 75 vs. FUS+TMZ | 0.0023 |
| TMZ 100 vs. FUS+TMZ | 0.3916 |
