# Supplementary material for: Focused Ultrasound-Induced Blood–Brain Barrier Opening to Enhance Temozolomide Delivery for Glioblastoma Treatment: A Preclinical Study
Source: PLoS One. 2013 Mar 19;8(3):e58995. doi: 10.1371/journal.pone.0058995 (PMC3602591; doi:10.1371/journal.pone.0058995)
Supplement: Table S3 — M-Cox proportional hazard model analysis when using FUS/TMZ as a reference. (DOCX) [file pone.0058995.s006.docx]

**Table S3. M-Cox proportional hazard model analysis when using FUS/TMZ as a reference.**

| Group | Median Survival (day) | p value | Hazard ratio (95% CI) |
| --- | --- | --- | --- |
| Control | 20 | 0.0009 | 8.987 (2.447) |
| TMZ, 50 mg | 20.5 | 0.0312 | 3.501 (1.120-10.944) |
| TMZ, 75 mg | 20 | **0.0039** | 6.080 (1.784-20.725) |
| TMZ, 100 mg | 19.5 | 0.0004 | 10.127 (2.802-36.596) |
| FUS/ TMZ | 23 | **(reference)** | 1.0 |
